# Barriers and Facilitators to Mental Health Support Among Airline Pilots: A Narrative Review

**DOI:** 10.7759/cureus.91340

**Published:** 2025-08-31

**Authors:** Piercarlo Minoretti

**Affiliations:** 1 Occupational Health, Studio Minoretti, Oggiono, ITA

**Keywords:** airline pilots, aviation safety, barriers, facilitators, mental health, occupational health

## Abstract

Mental health challenges among commercial air transport (CAT) pilots represent a significant concern for both individual well-being and aviation safety. Understanding the factors that facilitate or hinder access to appropriate psychological support within this occupational group is essential for the development of targeted and effective interventions. This narrative review was therefore undertaken to appraise and synthesize existing evidence on the barriers and facilitators influencing mental health care utilization among CAT pilots. A literature search was conducted across major academic databases, regulatory archives, and aviation-specific sources for English-language studies published between 2010 and 2025 that addressed barriers and facilitators to mental health help-seeking within the CAT pilot population. Eligible studies were thematically analyzed to identify key determinants of mental health support, with particular attention to help-seeking behaviors, organizational structures, and regulatory frameworks. The synthesis identified several primary barriers, including: (1) a prevailing culture of silence and stigma; (2) fears related to job security and medical certification; (3) limited trust in confidentiality; (4) regulatory and procedural constraints; and (5) organizational and logistical barriers. Conversely, key facilitators of mental health support comprised: (1) structured peer-support programs; (2) non-punitive disclosure and reporting policies; (3) comprehensive mental health education and initiatives to shift organizational culture; (4) accessible, independent, and confidential mental health care services; and (5) socioeconomic protections to mitigate perceived risks of disclosure. In summary, addressing mental health challenges among CAT pilots necessitates coordinated action across cultural, organizational, and regulatory domains. The implementation of confidential, non-punitive support mechanisms, in which occupational medicine plays a central role in prevention, early detection, and care coordination, combined with meaningful regulatory reform and sustained cultural change, holds the potential to significantly improve pilot well-being while strengthening overall aviation safety.

## Introduction and background

Commercial air transport (CAT) pilots operate within high-stakes occupational environments characterized by irregular schedules, complex decision-making responsibilities, and rigorous regulatory oversight [1]. Within this demanding operational context, mental health and psychological well-being have increasingly emerged as fundamental components of safe flight operations [2-7]. Despite this recognition, the aviation industry has historically exhibited cultural tendencies that can stigmatize psychological vulnerability and may, in turn, discourage help-seeking behaviors among pilots [8,9]. The consequences of this cultural paradigm have proven devastating, as evidenced by pilot suicides (also termed *aircraft-assisted suicides*) in high-profile incidents such as LAM Mozambique Airlines Flight 470 and Germanwings Flight 9525 - which exposed critical gaps in existing mental health support systems [10]. In response to such tragedies, current regulatory and organizational approaches continue to emphasize fitness-for-duty assessments designed to identify and remove pilots deemed psychologically unfit for service [11]. Paradoxically, however, this punitive model can create fundamental barriers to help-seeking, as pilots may rationally avoid disclosing mental health concerns due to legitimate fears of license suspension or career termination [4]. These systemic vulnerabilities became further amplified during the COVID-19 pandemic, when pilots faced unprecedented stressors - including rapidly evolving safety protocols, industry-wide instability, and widespread job insecurity - that intensified calls for comprehensive mental health reform within the aviation industry [12,13].

While existing literature has identified numerous hurdles that impede pilot access to psychological support [2,4,6,7], emerging research suggests that specific enablers may effectively promote help-seeking behaviors. Nevertheless, limited synthesis exists regarding how these barriers and facilitators interact within aviation contexts - particularly concerning the potential strategic integration of cultural, organizational, and regulatory interventions. To address this knowledge gap, we conducted a narrative review synthesizing available evidence around three critical research questions: What are the primary barriers preventing CAT pilots from accessing mental health support? Which facilitators have demonstrated promising effectiveness in overcoming these barriers? How can multilevel interventions be strategically coordinated to create more supportive occupational environments? Addressing these questions is essential for advancing both pilot wellbeing and aviation safety. As the industry continues to evolve, implementing evidence-based, preventive mental health strategies will become increasingly critical for ensuring safe and sustainable flight operations while supporting the psychological well-being of aviation professionals.

## Review

Methodology

A narrative review was conducted through searches of PubMed, PsycINFO, Web of Science, and Google Scholar electronic databases. Additional aviation-specific resources were consulted, including regulatory databases from the Federal Aviation Administration (FAA) [14] and the European Union Aviation Safety Agency (EASA) [15]. The literature review comprised original research articles, reviews, meta-analyses, systematic reviews, and regulatory documents published in English-language sources between January 1, 2010, and June 30, 2025. Selection criteria specifically targeted studies addressing mental health barriers and facilitators among CAT pilots, with emphasis on help-seeking behaviors, organizational support systems, and regulatory frameworks (Table 1).

**Table 1:** Search strategy and keywords for literature review on pilot mental health barriers and facilitators.

Concept domain	Search terms	Variations and synonyms
Population	Pilot*	"Pilot*", "Flight crew", "Commercial pilot*", "Airline pilot*"
-	Aviation	Airline*, Aeronautic*, "Air transport*", "Commercial aviation", "Civil aviation"
Mental Health	"Mental health"	"Psychological wellbeing", "Psychological health", "Mental wellbeing", Psychiatr*, Psycholog*
-	Depression	"Depressive disorder*", "Major depression", "Mood disorder*"
-	Anxiety	"Anxiety disorder*", Stress, "Psychological stress", "Occupational stress"
-	Suicide	"Suicidal ideation", "Self-harm", "Suicidal behavior*"
Barriers	Barrier*	Obstacle*, Impediment*, Challenge*, "Structural barrier*"
-	Stigma	"Mental health stigma", "Self-stigma", "Public stigma", "Professional stigma"
-	Fear	"Fear of consequences", "Career concerns", "Job security", "License revocation"
Facilitators	Facilitator*	Enabler*, "Promoting factor*", "Supportive factor*"
-	Support	"Support system*", "Social support", "Organizational support", "Peer support"
-	Program*	Intervention*, "Mental health program*", "Employee assistance", "Wellness program*"
Help-seeking	"Help-seeking"	"Help seeking", "Treatment seeking", "Service utilization", "Healthcare utilization"
-	Access	"Access to care", "Service access", "Treatment access", "Mental health service*"

**Figure 1:**
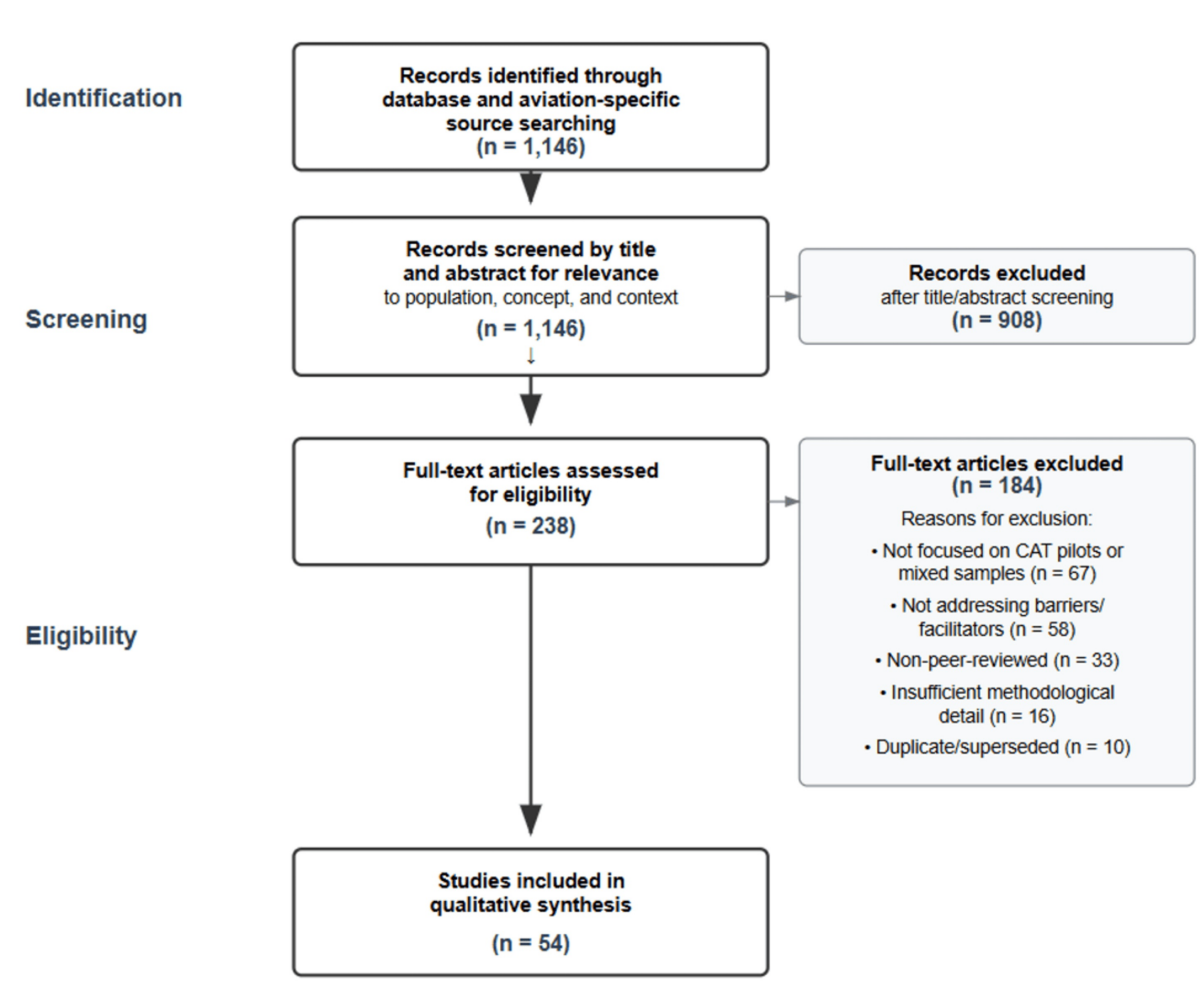
Study selection process.

Excluded were conference abstracts, case reports, and non-peer-reviewed materials (e.g., preprints). A total of 1,146 records were initially identified across databases and aviation-specific sources. Titles and abstracts were screened for relevance to the population (commercial air transport pilots), concept (barriers/facilitators to mental health help-seeking or support), and context (occupational, organizational, and regulatory settings), resulting in 238 records advanced to full-text assessment. Full-text review excluded 184 records for the following common reasons: not focused on CAT pilots or mixed samples without pilot-specific results (*n* = 67); not addressing barriers/facilitators or help-seeking/support utilization (n = 58); non-peer-reviewed sources (*n* = 33); insufficient methodological detail for appraisal (*n* = 16); duplicate or superseded reports (*n *= 10). Fifty-four documents met the inclusion criteria for qualitative synthesis (Figure 1).

**Figure 2:**
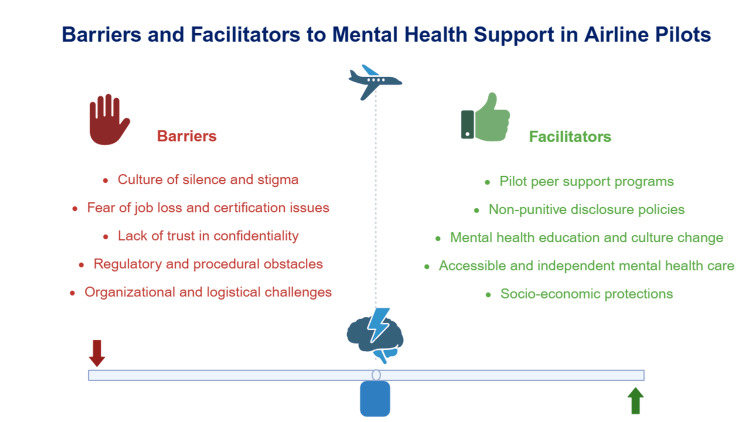
Conceptual overview of barriers and facilitators to mental health support utilization among commercial air transport pilots. Image credits: Piercarlo Minoretti.

Given the narrative design, no quantitative meta-analysis was performed. Barriers and facilitators to mental health help-seeking among pilots were identified through a thematic synthesis of the available data (Figure 2). 

Results

Barriers to Mental Health Support Among Airline Pilots

Culture of silence and stigma:* *The aviation industry has historically fostered an occupational culture emphasizing self-reliance and unwavering professional competence [16]. Although these characteristics may offer functional advantages in high-stakes flight operations, they can also inadvertently hinder the recognition, disclosure, and effective management of mental health concerns. Stigma and limited mental health awareness remain relatively common, frequently contributing to a tendency toward silence around psychological distress [4,17,18]. Notably, within the aviation sector, stigma may manifest through multiple, interrelated layers of influence. At the individual level, CAT pilots may internalize professional norms that characterize psychological difficulties as evidence of personal inadequacy and occupational unfitness [19]. Accordingly, epidemiological data indicate that the prevalence of major depression among pilots (1.9%-12.6%) approximates that of the general population [3, 10]; however, disclosure rates remain disproportionately low [8]. At the interpersonal level, peer dynamics may encourage concealment, as empirical evidence indicates that pilots tend to exhibit overconfidence in assessments of their own mental health while expressing comparatively limited confidence in the mental health of their colleagues [20]. Organizationally, institutional policies may position mental health disclosure as a liability rather than a routine, non-punitive component of occupational health management [21]. The multifaceted consequences arising from these dynamics have been the subject of growing scholarly attention, with an expanding body of empirical evidence documenting their mechanisms and implications. In this regard, studies employing anonymous reporting methodologies have tended to identify higher rates of depressive symptoms than those reported through regulated aeromedical examinations, suggesting that formal assessment channels may not fully capture their true prevalence or impact [7,8,22]. Such findings lend support to the interpretation that pilots may engage in deliberate impression-management strategies, concealing symptoms and avoiding settings in which formal psychological evaluation is conducted. As a result, the responsibility for managing mental health is disproportionately borne by individuals who may concurrently experience significant professional and personal repercussions when their well-being deteriorates.

Fear of job loss and certification issues: Across multiple jurisdictions, regulatory frameworks designate certain mental health conditions as potential grounds for suspension or revocation of the medical certificate required for licensure [23]. For numerous pilots, such provisions constitute not merely a professional hurdle but an existential threat to their occupational identity and economic stability. Consequently, the prospect of career‑related repercussions appears to be one of the most significant deterrents to help‑seeking among CAT pilots [4,21,24]. Notably, in a recent qualitative phenomenological study of airline pilots (*n* = 21), every participant expressed hesitation to disclose psychological symptoms because they believed disclosure could jeopardize their ability to maintain certification, despite formal assurances and the existence of support resources [4]. In addition, participants frequently referenced colleagues who had lost their licenses and sustained substantial financial harm following disclosure, perpetuating a culture of caution and neglect [4]. In a study of U.S. civilian pilots (*n* = 3,765), 56.1% of the study participants reported healthcare avoidance due to fear of losing aeromedical certification, 26.8% misrepresented/withheld information on health questionnaires for the same reason, and 45.7% sought informal care [21]. In a separate investigation of Canadian pilots (*n *= 1,405), 72% worried about seeking medical care due to career impact, and 46% avoided or delayed care for a symptom [24]. Structural and procedural features of regulatory systems appear to further entrench these tendencies. Accordingly, disclosure of a mental health condition typically initiates protracted review processes during which flight privileges remain suspended - a condition that is commonly perceived as both professionally and financially destabilizing [25]. These disincentives are further compounded by time‑based regulatory criteria. In this regard, under FAA policy, pilots treated with approved selective serotonin reuptake inhibitors - a class of antidepressant drugs - must demonstrate six months of symptom-free stability on a constant dose before initial special-issuance review [26], typically resulting in extended grounding during this period; comparable stability and assessment requirements in UK/EASA guidance may produce multi-month suspensions until fitness is re-established. Unfortunately, such burdens do not operate in isolation but may compound with broader industry stressors - particularly during crises such as the COVID‑19 pandemic, when financial instability emerged as a significant driver of stress‑related morbidity [27]. This convergence produces a critical paradox: when seeking mental health treatment carries a plausible risk of jeopardizing certification, pilots may rationally, though detrimentally, opt to forgo engagement with mental health professionals, even when such care has the potential to reduce operational risk.

Lack of trust in confidentiality: Distrust regarding the confidentiality of mental health services constitutes another significant barrier to help‑seeking. In the study by Cross et al. [4], 81% of the study participants reported unwillingness to use employer‑provided or regulator‑sponsored resources due to concerns that such use could prompt regulatory disclosure and jeopardize medical certification, indicating that distrust is both prevalent and consequential. This pervasive skepticism can reflect a confluence of objective structural constraints and subjective perceptions that together depress utilization of available support. Structurally, confidentiality protections within aviation‑specific mental health services are more restrictive than in typical clinical settings [4,9]. In this regard, under different regulatory provisions, providers may be subject to mandatory reporting to Aviation Medical Examiners (AMEs) or civil aviation authorities under defined circumstances, and federal rules require applicants to disclose all mental health treatment within the prior three years on medical certification applications [28] - possibly eroding long‑term confidentiality protections irrespective of outcomes or provider assurances. The interplay of medical privacy regulation and aviation safety oversight produces complex and, at times, ambiguous reporting obligations that pilots frequently interpret as threats to professional security [29]; even where legal safeguards exist, uncertainty about their scope and limits may foster assumptions that information shared with licensed clinicians may ultimately be transmitted to regulatory authorities [30]. Industry policies can further complicate this landscape. In this regard, some operators require pilots to report mental health treatment to management as a condition of employment, expanding disclosure pathways beyond those strictly mandated by regulation and amplifying perceptions of risk [4,31]. The organizational affiliation of certain services with employers or aviation authorities can heighten distrust, as pilots may infer that participation might invite additional scrutiny and threaten professional standing [16]. Although AMEs may act in pilots’ best interests, their mandated role in regulatory compliance is commonly perceived as incompatible with strict confidentiality, potentially reinforcing avoidance of formal services and channeling pilots toward informal or unregulated care that circumvents documentation and oversight [4, 29]. Collectively, these dynamics undermine organizational and regulatory mental health initiatives and may impede timely identification and early intervention.

Regulatory and procedural obstacles: The regulatory environment governing pilot mental health is still commonly perceived as punitive rather than therapeutically supportive, with processes that deter proactive care [18]. Importantly, following disclosure through official channels, pilots frequently face bureaucratic hurdles that can prolong grounding beyond clinical necessity, ultimately amplifying psychological and financial strain [6]. Deferred cases may require months for federal review, leaving pilots unable to exercise privileges while fostering uncertainty. Return-to-duty procedures can also demand multilayered specialist evaluations from board-certified psychiatrists, neuropsychologists, and designated aviation medical examiners before formal review commences [32]. These requirements are routinely described as onerous and expensive, operating as critical structural barriers to both recertification and upstream care-seeking. Administrative inefficiencies may further compound these challenges, as delayed document intake extends processing timelines and obscures case status, thereby undermining both transparency and predictability [33]. In this context, procedural practices continue to impede adequate mental health care, with medication policies exemplifying a sustained misalignment. Specifically, antidepressant stabilization rules frequently require months of documented stability before recertification, regardless of individual recovery trajectories [26] - a conservative stance that, though aimed at mitigating acute risk, may paradoxically increase long‑term safety risks by suppressing early detection and treatment.

Organizational and logistical challenges: The occupational profile of CAT pilots - characterized by irregular scheduling, high geographic mobility, fatigue, circadian disruption, and time constraints - can systematically impede the establishment and continuity of care that is essential for effective mental health support [34]. Irregular rosters, often comprising extended duty periods and unpredictable assignments, further complicate coordination with mental health providers and make it challenging to sustain consistent engagement in treatment [6]. Limited ability to confirm availability beyond a few days in advance may also undermine continuity, particularly with specialized clinicians who schedule far in advance. Given the central role of stable therapeutic engagement in achieving optimal clinical outcomes [35], scheduling volatility can significantly undermine both access to care and the overall effectiveness of treatment. Geographic mobility may further disrupt care continuity across pilots’ career lifecycles. Moreover, base changes resulting from seniority bidding, fleet transitions, mergers, and career advancement often require pilots to navigate unfamiliar healthcare systems, reestablish relationships with providers, and adapt to regional network variations, all of which can disrupt continuity of care and complicate sustained engagement [36]. Extended duty cycles, night operations, and transmeridian travel can also disrupt sleep, impair cognitive performance, and diminish motivation, reducing the likelihood that pilots will prioritize appointments during limited off‑duty periods [37]. This situation creates a paradox in which the very operational stressors that heighten mental health needs also suppress treatment‑seeking and adherence. Time constraints during compressed rest intervals can further exacerbate these challenges, as CAT pilots must balance family responsibilities, mandated rest periods, and recurrent training requirements [38], leaving mental health care to compete with other non‑deferrable obligations. As a result, cancellations and postponements become common, interrupting treatment trajectories and eroding therapeutic progress.

Facilitators of Mental Health Support Among Airline Pilots

Pilot peer support programs: Pilot peer support programs (PPSPs) have emerged as one of the most promising facilitators for mental health support in commercial aviation [16,39,40]. These programs leverage the unique understanding, credibility, and cultural competence that pilots possess when supporting their peers, creating accessible, non-threatening pathways to psychological support that circumvent traditional barriers [40]. In structured PPSPs, trained pilot volunteers provide confidential listening, emotional support, practical guidance, and professional referral services to colleagues experiencing personal or professional challenges [18]. The effectiveness of peer support programs stems from two main interconnected factors that address core barriers to help-seeking in aviation. First, peer supporters possess an intimate understanding of the unique occupational stressors inherent to CAT, including irregular scheduling, circadian disruption, geographic displacement, regulatory pressures, and the psychological demands of high-stakes decision-making [41]. This specialized knowledge enables them to relate authentically to concerns about career impact, certification issues, and the complex interplay between personal well-being, mental health, and professional obligations. Second, well-designed PPSPs operate under strict confidentiality protocols with clearly defined boundaries that protect participants from involuntary disclosure to management or regulatory authorities [18,29]. Pilot peer support programs that have demonstrated effectiveness consistently share a core set of design principles. First, peer supporters typically undergo structured training to develop essential competencies, including active listening, psychological first aid and crisis management, mental health literacy, maintenance of professional boundaries, and recognition of aviation‑specific stressors [18]. Second, program operations are supported by clearly articulated procedural safeguards, most notably well‑defined confidentiality standards and structured escalation pathways [42]. These safeguards are further reinforced through formal referral mechanisms that ensure timely access to qualified mental health professionals when clinical intervention is required. Finally, although these elements represent foundational features of effective programs, the specific operational details - including training requirements, continuing education provisions, and the scope of referral networks - must be adapted to the organizational and regulatory contexts in which the programs are implemented [18,42].

Non-punitive disclosure policies: Non‑punitive disclosure policies in civil aviation mark a shift from punitive deterrence to evidence‑informed support, using confidential, peer‑led pathways to encourage early help‑seeking by pilots while avoiding automatic loss of medical certification or employment where safety permits [18,39]. Such an approach recognizes that mental health challenges are frequently temporary and highly treatable conditions [43] and that supporting pilots through psychological difficulties may better serve aviation safety than summarily removing them from service - except in cases demonstrating imminent safety risk based on objective clinical assessment rather than diagnostic categories alone. In this scenario, operators may distinguish between different types and severities of mental health conditions - applying graduated responses proportionate to actual functional impairment rather than employing blanket policies that treat a specific diagnosis as uniformly disqualifying [9,31]. In this context, aeromedical policies may employ conditional certification mechanisms - such as limitations, time-limited or special-issuance certificates, and monitored treatment-and-follow-up protocols - rather than immediate suspension or revocation [28]. In addition, occupational health physicians may operationalize these conditional pathways by conducting integrated evaluations of physical and psychological fitness, determining functional limitations, and recommending graded duty modifications matched to the pilot’s current capacity. Examples include temporary restrictions on night or ultra‑long‑range operations, paired flying with enhanced oversight, adjusted scheduling to mitigate fatigue, or time‑limited administrative/training assignments during treatment stabilization and follow‑up. Such physician‑guided adaptations prioritize safety while preserving work identity and continuity of employment, reserving full removal from flight duties for circumstances that present imminent risk to self or others based on objective clinical assessment. Taken together, these approaches can maintain pilots’ standing within the certification framework and a defined pathway back to flying while addressing health needs through structured evaluation and treatment. Notably, emerging evidence suggests that confidential, non-punitive disclosure frameworks can be associated with increased pilot willingness to proactively address mental health concerns [4,18].

Mental health education and culture change: Industry-wide efforts to educate CAT pilots and aviation professionals about mental health are catalyzing significant cultural attitude shifts that address foundational stigma and misconceptions that have historically deterred help-seeking [44]. These comprehensive initiatives may include mental health awareness training, systematic integration of psychological well-being topics into initial and recurrent pilot training curricula, and high-profile de-stigmatization campaigns led by aviation authorities, professional organizations, and airline leadership [45]. In general, effective education programs are designed to transcend simple information dissemination about mental health conditions and symptoms, instead addressing aviation-specific concerns that resonate with pilot experiences and priorities [46]. These programs explicitly clarify regulatory processes, timelines, and requirements; destigmatize help-seeking behaviors; and provide practical, actionable resources for accessing appropriate support. Programs incorporating testimonials from pilots who have successfully navigated mental health challenges and returned to full flying status may prove particularly impactful in demonstrating that career recovery is not only possible but common when appropriate support is accessed early [47]. In addition, comprehensive culture change efforts can extend substantially beyond formal educational initiatives to comprise organizational messaging and leadership behavior modeling that reinforces supportive attitudes toward mental health [20,31].

Accessible and independent mental health care: The availability of mental health care that is simultaneously accessible to pilots and operationally independent from airline or regulatory oversight represents a crucial facilitator that addresses multiple barriers to help-seeking within a single intervention framework [5]. Innovative programs should increasingly guarantee confidential access to mental health providers who possess a deep understanding of aviation culture and stressors while operating with complete independence from any reporting obligations to employers, regulatory authorities, or insurance carriers [6,18]. These models commonly involve carefully selected third-party providers specifically contracted to serve pilot populations while maintaining strict confidentiality boundaries that exceed traditional healthcare privacy protections - unless specific, predetermined safety thresholds that indicate imminent risk of harm are met [9]. Clear, transparent communication about these confidentiality boundaries, their scope, limitations, and exceptions helps build trust and encourages utilization among pilots who might otherwise avoid formal mental health services [48]. Accessibility features specifically designed to facilitate pilot engagement can also address the unique logistical challenges inherent to commercial aviation careers [49]. For example, high-profile telehealth or artificial intelligence-driven options may enable continuity of care for pilots away from home base or facing geographic relocations [50]. These virtual care platforms should be designed to include secure messaging, crisis support features, and scheduling systems designed specifically for mobile professionals.

Socioeconomic protections: Financial security during periods of medical grounding represents a fundamental facilitator for mental health help-seeking that directly addresses one of the most significant barriers deterring pilots from accessing appropriate care [51]. Pilots with robust disability insurance coverage, comprehensive airline policies providing income protection during mental health treatment, or alternative duty assignments during recovery report substantially greater willingness to disclose psychological concerns and engage with therapeutic services [52]. These protections may address the core economic fears that systematically discourage help-seeking even when pilots recognize the need for support. In addition, certain collective-bargaining agreements and advanced pilot-support frameworks can stipulate that flight crew members on authorized medical or disability leave continue to accrue seniority, thereby preserving their bid position and upgrade eligibility throughout treatment and convalescence [45]. Alternative duty pathways can enable pilots to contribute meaningfully to organizational operations through training roles, safety positions, or administrative functions while maintaining income and professional engagement during periods when flying duties are contraindicated [47]. State-of-the-art policies should recognize that mental health recovery may occasionally require career transition or retraining, and provide supportive benefits including educational assistance, career counseling, and transition support for pilots who may need to pursue alternative career paths [6]. Notably, the costs associated with providing income protection and career continuity during mental health treatment are often substantially lower than expenses related to pilot replacement, retraining, and the operational disruptions associated with unexpected worker unavailability [53]. Moreover, pilots who receive appropriate support during mental health challenges often return to service with enhanced resilience, improved stress management capabilities, and greater organizational loyalty [54].

Discussion

The complex ecosystem of barriers and facilitators affecting CAT pilot mental health support identified in the current review revealed significant contradictions within current aviation safety paradigms - underscoring the urgent need for systematic reform that transcends traditional approaches to occupational health management in safety-critical industries. Importantly, the intricate interplay between individual, organizational, and regulatory factors clearly suggests that addressing pilot mental health requires coordinated intervention across multiple domains rather than isolated initiatives [55]. At the core of these findings lies a profound paradox that challenges foundational assumptions about safety management. In this scenario, systems ostensibly designed to ensure aviation safety may paradoxically compromise it by creating major barriers to help-seeking that incentivize concealment of treatable mental health conditions. When pilots perceive that acknowledging psychological concerns poses an existential threat to their professional livelihood and economic security, they can become systematically incentivized to conceal problems that could be successfully addressed through appropriate clinical intervention. This dynamic creates a hidden risk reservoir within the aviation system, where untreated mental health issues may progress to more serious psychological disturbances that could compromise operational safety and decision-making capacity [56]. Expectedly, non-punitive frameworks that support pilots through mental health challenges while maintaining appropriate clinical safeguards appear to offer superior outcomes for both individual well-being and overall system safety [2,18], fundamentally challenging the traditional binary approach that characterizes psychological conditions as either completely disqualifying or entirely irrelevant to operational capacity. Cultural transformation emerged as equally critical to structural changes in regulations and organizational procedures, highlighting that common stigma surrounding psychological distress in aviation cannot be adequately addressed through administrative reform alone but requires sustained efforts to shift professional norms, peer attitudes, and individual beliefs about mental health and help-seeking behaviors - possibly through multiple reinforcing elements [20]. Intriguingly, the most effective facilitators identified in the current review were consistently those addressing multiple barriers simultaneously through integrated, systems-based approaches. PPSPs exemplify this aspect by combining trusted peer relationships leveraging shared occupational experiences, robust confidentiality protections addressing disclosure concerns, and established referral pathways to independent clinical care - thereby simultaneously addressing stigma, trust, and accessibility barriers [39-42]. Similarly, an integrated suite of regulatory interventions that combines protected-disclosure mechanisms with socioeconomic safeguards is poised to mitigate pilots’ fundamental apprehensions about career disruption and financial repercussions through a multifaceted approach. 

Beyond regulatory and organizational levers, occupational medicine represents a fundamental, practice-proximal intervention mechanism for facilitating safe, early, and non-punitive care delivery. Through this framework, occupational health physicians may systematically evaluate physical and psychological status, calibrate work demands with current functional capacity through graduated duty modifications, and coordinate timely referrals to specialized psychological support services. Concurrently, these practitioners may implement comprehensive prevention strategies comprising mental health literacy education, fatigue mitigation protocols, and stigma reduction interventions. Building upon these preventive measures, they can also systematically address psychosocial risk determinants while conducting periodic fitness-for-duty assessments that support structured return-to-work pathways. The strategic integration of occupational medicine within peer support networks, protected-disclosure mechanisms, and conditional-certification frameworks subsequently creates a unified approach that synthesizes clinical oversight with workplace adaptation strategies, ultimately improving both care engagement and operational safety outcomes.

Despite growing recognition of pilot mental health as a critical aviation safety issue, significant knowledge gaps persist that limit the development of evidence-based interventions. The actual prevalence of mental health conditions among pilots remains poorly characterized due to systematic underreporting and the absence of comprehensive epidemiological studies accounting for concealment behaviors incentivized by current structures. Intervention effectiveness, while demonstrating promise in initial implementations, requires more rigorous evaluation through controlled studies and systematic outcomes assessment to establish best practices replicable across diverse aviation contexts. Furthermore, research and policy development have focused primarily on CAT pilots within Western frameworks, leaving uncertainty regarding applicability to other aviation sectors and cultural contexts. Gender differences in help-seeking barriers and intervention effectiveness also warrant investigation, given increasing workforce diversity and evidence suggesting that traditional approaches may not adequately address the distinct needs of female pilots and other underrepresented groups.

## Conclusions

The evolution of pilot mental health support systems has recently reached a critical juncture, which creates unprecedented potential for comprehensive reform that could fundamentally transform aviation’s approach to psychological wellbeing while enhancing operational safety. Within this scenario, major coordination efforts are required across multiple stakeholders - including regulatory authorities developing policies balancing safety oversight with pilot wellbeing, airline management investing in comprehensive support systems, pilot unions advocating for member wellbeing while shifting cultural attitudes, mental health providers developing aviation-specific expertise, and innovative service delivery models (including telehealth). The stakes extend beyond individual pilot welfare to affect passenger safety, airline economic viability, and broader social acceptance of mental health as legitimate healthcare deserving equal attention and resources as physical conditions. The ultimate goal - skies that are safe because pilots are holistically healthy and supported - is not only achievable but essential for the future of aviation.
